# *Aquirufa lenticrescens* sp. nov. and *Aquirufa aurantiipilula* sp. nov.: two new species of a lineage of widespread freshwater bacteria

**DOI:** 10.1007/s00203-022-02950-6

**Published:** 2022-06-02

**Authors:** Alexandra Pitt, Ulrike Koll, Johanna Schmidt, Meina Neumann-Schaal, Jacqueline Wolf, Sophia Krausz, Martin W. Hahn

**Affiliations:** 1 Research Department for Limnology, University of Innsbruck, Mondseestrasse 9, 5310 Mondsee, Austria; 2 Chemical Analytics and Metabolomics, Leibniz Institute DSMZ-German Collection of Microorganisms and Cell Cultures GmbH, Brunswick, Germany

**Keywords:** *Aquirufa*, *Cytophagaceae*, Freshwater, Genome, Genome size

## Abstract

Two bacterial strains, 9H-EGSE^T^ and 15D-MOB^T^, were isolated from small freshwater habitats located near Salzburg, Austria. They showed the highest 16S rRNA sequence similarities of 100% and 99.9%, respectively, with type strains of species of the genus *Aquirufa* (*Bacteroidota*). Genome-based phylogenetic reconstructions with 119 amino acid sequences assigned the new taxa to the two distinct branches of the genus *Aquirufa*. Whole-genome average nucleotide identities were calculated with all possible pairs belonging to the genus. Values between 75.4% and 88.6% revealed that the two new strains represent each a new species. Like all, so far described members of the genus, they grew aerobically and chemoorganotrophically, were rod-shaped, red-pigmented, and motile by gliding, and showed genome sizes of about 3 Mbp and G + C values of about 40%. They could be distinguished by some phenotypic and chemotaxonomic features from their nearest related species. Until now, strain 9H-EGSE^T^ is the only one among the *Aquirufa* strains which contained traces of MK8 as respiratory quinone, and strain 15D-MOB^T^ is the only one that formed tiny orange globules in liquid medium. The genome of strain 9H-EGSE^T^ comprised genes for the complete light-harvesting rhodopsin / retinal system, in the case of 15D-MOB^T^ genes predicted for a nitrous oxide reductase were present. For the two new species of the genus *Aquirufa*, we propose to establish the names *Aquirufa lenticrescens* for strain 9H-EGSE^T^ (= JCM 34077^ T^ = CIP 111926^ T^) and *Aquirufa aurantiipilula* for strain 15D-MOB^T^ (= JCM 34078^ T^ = CIP 111925^ T^).

**Supplementary Information:**

The online version contains supplementary material available at 10.1007/s00203-022-02950-6.

## Introduction

The genus *Aquirufa* was first described by Pitt et al. in 2019 (Pitt et al. 2019) and comprises at the time of writing five species (Pitt et al. 2019, 2020; Sheu et al. 2020). The genera *Aquirufa* and *Sandaracinomonas*, described in 2020 (Chen et al. 2020), formed a lineage closely related to the genera *Flectobacillus*, *Arcicella,* and *Pseudarcicella*. The five genera of this clade were accommodated in the family *Cytophagaceae* (Skerman et al. 1980), belonging to the phylum *Bacteroidota* (Oren and Garrity 2021). Since genome-based phylogenetic analyses revealed that the family *Cytophagaceae* is non-monophyletic (Hahnke et al. 2016; García-López et al. 2019) García-López et al*.* proposed to split the *Cytophagaceae* into three distinct families. Besides *Cytophagaceae* and *Flexibacteraceae*, validated in 2020 (Oren and Garrity 2020b), they recommended using the validly published name *Spirosomaceae* proposed in 1978 (Larkin and Borrall 1978). At the time of establishment, the family included only the genera *Spirosoma*, *Flectobacillus*, and *Runella* (Larkin and Borrall 1978). In their emended description García-López et al. suggested adding 22 genera to the *Spirosomaceae*, this opinion was listed as a notification (Oren and Garrity 2020a) but is at the time of writing not yet validated. Nevertheless, while the three genera *Flectobacillus*, *Arcicella*, and *Pseudarcicella* were proposed for assignment to the *Spirosomaceae*, this would have to apply as well for the later described and rather closely related genera *Aquirufa* and *Sandaracinomonas*, which form a clade together with these three genera (Pitt et al. 2019).

**Fig. 1:**
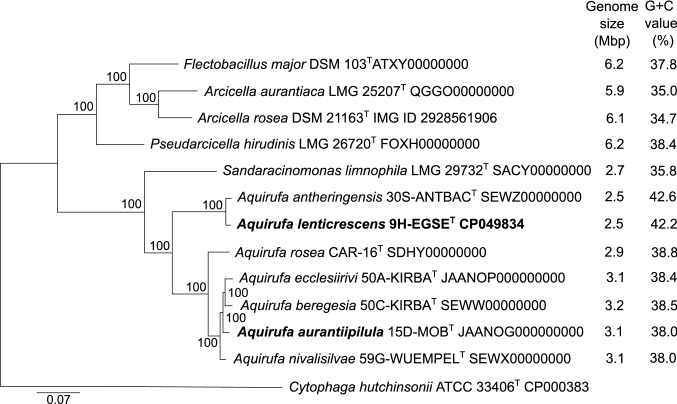
Phylogenomic RAxML tree calculated with amino acid sequences obtained from 119 single-copy genes from all available genomes of the taxa from Fig. 4. Bar, 0.07 substitutions per nucleotide position

All by now, described members of this clade, as well as the new strains of this study, have in common that they were isolated from aquatic habitats or sources associated with water (Kämpfer et al. 2012; Chen et al. 2013, 2020; Sheu et al. 2017; Pitt et al. 2020). While the strains of the genera *Aquirufa* and *Sandaracinomonas* have genome sizes of about 3 Mbp, the available genome sequences of further species of the clade are double the size (Fig. 1).

The genus *Aquirufa* is characterized by aerobic, chemoorganotrophic, rod-shaped, red-pigmented, and motile bacteria (Pitt et al. 2019). They seem to be widespread (Pitt et al. 2019) and sometimes abundant (Cruaud et al. 2020) and occur in a broad range of standing and running freshwater habitats (Cruaud et al. 2020; Pitt et al. 2019, 2020). Because the interspecific 16S rRNA gene sequence similarities of type strains are near 100% or exact 100%, the genus *Aquirufa* represents a cryptic species complex, which makes it impossible to assign publicly available 16S rRNA sequences of uncultured bacteria to particular *Aquirufa* species (Pitt et al. 2020). Nevertheless, there seemed to be an imbalance in the distribution of the members of the two main branches of the genus *Aquirufa* (Fig. 1). The NCBI Nucleotide BLAST website (Johnson et al. 2008) was used to search for matches for the 16S rRNA gene sequences similarities of ≥ 99.0%, considering isolates and environmental samples. A lot of detections affiliated with the ‘*Aquirufa antheringensis* branch’ (strain 30S-ANTBAC^T^) were found in studies all over the world at various freshwater habitats. Some examples, which show the broad range of habitats are temperate rivers in Massachusetts (USA) (Crump and Hobbie 2005), Tibetan lakes (Zhang et al. 2013), Canadian permafrost thaw ponds (Rossi et al. 2013), freshwater lakes in Japan (Kojima et al. 2014), in Europe beside Austria, a mountain lake in France (Biderre-Petit et al. 2011), a drinking water reservoir in Greece (Lymperopoulou et al. 2012), and Karst cave water pools in Switzerland (Shabarova et al. 2013). Surprisingly, corresponding searches with the sequence of strain 59G-WUEMPEL^T^ (‘*Aquirufa nivalisilvae* branch’) revealed fewer detections, even though, this branch contains five species, while the ‘*Aquirufa antheringensis* branch’ comprises only two species, in both counting are the two new strains described here already included.

In this study, we present the description of two additional members of the genus *Aquirufa*. For this purpose, we sampled various freshwater habitats and inoculated agar plates with prefiltered water samples. We used the characteristic red pigmentation of the colonies to search for strains potentially belonging to the target group. Among the obtained strains were two, 9H-EGSE^T^ and 15D-MOB^T^, which each represent a new species for which we propose the names *Aquirufa lenticrescens* and *Aquirufa aurantiipilula*, respectively.

## Materials and methods

### Home habitats and isolation

Strain 9H-EGSE^T^ originated from Grossegelsee, a small natural pond located near the town of Mattsee, Austria. The water sample was taken from the lakeside in July 2019 at the approximate geographic coordinates 47.9622 N and 13.1245 E. Further sampling in July 2021 revealed pH 8.3 and conductivity of 471 µS cm^−1^. Strain 15D-MOB^T^ originated from Moosbach, a small creek running through Mondsee, a village located near Salzburg, Austria. The water sample was taken in July 2019 at the approximate geographic coordinates 47.8616 N and 13.3399 E. Further sampling in July 2021 revealed pH 8.1 and conductivity of 619 µS cm^−1^. The isolation procedure aimed to obtain strains belonging to the genus *Aquirufa*. For that purpose, liquid nutrient broth, soytone, yeast extract (NSY) (Hahn et al. 2004) medium and NSY agar plates were used. The water sample of the pond Grossegelsee was filtered through a 0.65 µm pore size filter and the filtrate was subsequently spread on agar plates. The water sample of the creek Moosbach was treated in the same way, but using a 0.8 µm pore size filter. After one week of incubation at room temperature, small portions of colonies with the characteristic red pigmentation of *Aquirufa* strains were picked and transferred to 24-well plates filled with liquid NSY medium. These cultures were tested by partial sequencing of 5’-end of the 16S rRNA genes (primer pair 27F, 1492R) and comparative sequence analyses for affiliation with the genus *Aquirufa.* Promising cultures were subsequently purified. Because the 16S rRNA gene sequence similarities between *Aquirufa* species are relatively high (Pitt et al. 2020), the 16S rRNA gene is not appropriate to identify potential new species. Therefore, analyses of the sequences of the marker gene *gyrB* encoding the B subunit of the DNA gyrase (primer pair ArciLike-gyrB-F2 5’-TGCATTTCAAGCTATCTTGCC-3’ and ArciLike-gyrB-R2 5’-ACTTCATCTCCCATCAACATC-3’) were used to reduce the number of cultures to presumable candidates for new species. Finally, these remaining strains were genome-sequenced and the following analyses (see below) served to identify strains, which represent new species.

### Phenotypic and chemotaxonomic characterization

The temperature range for growth was tested on a series of NSY agar plates exposed to increasing temperatures starting at 6 °C until no growth was observed. NaCl tolerance was tested using agar plates with various NaCl concentrations in 0.1% w/v steps. For testing anaerobic growth, an anaerobic chamber and standard NSY agar plates, as well as NSY plates supplemented with 2 g l^−1^ NaNO_3_, were used. For determination of cell morphology and cell dimension, well-growing liquid cultures were fixed with 2% paraformaldehyde, stained with DAPI (4′,6-diamidino-2-phenylindole), and investigated using an epifluorescence microscope (UV filter). Soft agar plates (1 g l^−1^ yeast extract, 0.1 g l^−1^ K_2_HPO_4_, and 2.0 g l^−1^ agar) were used to test the strain for motility (Hahn et al. 2017). One drop of a well-growing culture was placed in the center of these test plates, as well as on standard NSY plates, incubated at room temperature, and observed for several days. Assimilation of various substrates was tested using GEN III MicroPlates^T^ (Biolog), which detect utilization of substrates as electron donators by the subsequent reduction of a tetrazolium redox dye. Cells from well-growing liquid cultures were centrifuged and added to the inoculum medium so that the OD of the culture corresponded to 0.07 at a wavelength of 590 nm. After 48 h incubation at room temperature, the absorption was measured with a Multiskan FC (Thermo Scientific) at a wavelength of 595 nm. After subtracting the value of the negative control (without test substrate), obtained values from 0.016 to 0.05 were regarded as weak utilization, < 0.016 as negative, and > 0.05 as positive. The chemotaxonomic characterization included analyses of the composition of whole-cell fatty acids, polar lipids, and respiratory quinones. These analyses were carried out by the Identification Service, Leibniz Institute DSMZ-German Collection of Microorganisms and Cell Cultures GmbH. For all chemotaxonomic investigations, cells were inoculated into liquid NSY medium and harvested after 3 days of growth (room temperature) by centrifugation. For the whole-cell fatty acid composition, an Agilent Technologies 6890 N instrument and the Microbial Identification System (MIDI) Sherlock version 6.1 (method: TSBA6 database) were used as described by Sasser (Sasser 1990). The same extract was further analyzed by GC/MS to confirm peak identity and resolve summed features (Vieira et al. 2021). Double bond positions were analyzed by further derivatization to dimethyl disulfide adducts and subsequent GC/MS analysis (Moss and Lambert-Fair 1989). Polar lipids were extracted and analyzed as described by Tindall (Tindall 1990a, b) based on the method by Bligh and Dyer (Bligh and Dyer 1959). Polar lipids were separated by two-dimensional silica gel thin-layer chromatography. Total lipid material was detected using dodecamolybdophosphoric acid (Dmp) and specific functional groups by α-naphthol, ninhydrin, and molybdenum blue. Respiratory quinones were extracted by solid-phase extraction and analyzed by reversed-phase HPLC coupled to a diode array detector as described previously (Vieira et al. 2021).

### Genomic characterization

DNA extraction and genome sequencing were performed as described previously (Hoetzinger et al. 2017). A shotgun library was paired-end sequenced on an Illumina HiSeq instrument (2 × 150 bp). De novo genome assembly was performed using the software SPAdes version 3.13.0 (Bankevich et al. 2012) and resulted in the case of strain 9H-EGSE^T^ in a closed genome with a coverage value of 690x, with a size of 2.5 Mbp and in the case of strain 15D-MOB^T^ in 80 contigs with a coverage value of 500 × and a genome size of 3.1 Mbp. The obtained genome sequences were annotated by the NCBI Prokaryotic Genome Annotation Pipeline (Tatusova et al. 2016) and deposited at DDBJ/ENA/GenBank databases. For further comparative analyses, the genomes were also annotated by the Integrated Microbial Genomes & Microbiomes Expert Review (IMG/MER) system and incorporated into the IMG database (Chen et al. 2019). Homologous protein-encoding genes were identified using the IMG/MER (Chen et al. 2019) system (minimum 30% percentage identity, max. e-value 1e^−5^). The SEED viewer (Overbeek et al. 2005) was used for an amino acid sequence-based comparison of the new strains with their nearest relatives. Whole-genome average nucleotide identity (gANI) values and corresponding alignment fraction (AF) values were calculated for all possible pairs of the new strains with *Aquirufa* type strains using the IMG/MER system (Chen et al. 2019). In addition, digital DNA–DNA hybridization (dDDH) values were determined using the *Type (Strain) Genome Server* (Meier-Kolthoff et al. 2021).

### Phylogenetic reconstructions

Phylogenetic trees were calculated using almost full-length sequences of the 16S rRNA gene and genome-based, using amino acid sequences of 119 single-copy marker genes (Parks et al. 2018). For the phylogenetic tree based on 16S rRNA gene sequences, the software MEGA X (Kumar et al. 2018) was used. The sequences were aligned and analyzed for the best-fitting substitution model. This resulted in neighbor-joining, maximum likelihood and parsimony reconstructions with the parameters Kimura 2 model (Kimura 1980), gamma-distributed (5 categories), invariant sites, and 1000 bootstrap replicates. For the phylogenetic tree based on genome sequences, 119 amino acid sequences of 120 protein-encoding genes recommended by Parks et al*.* (Parks et al. 2018) were used. One gene (protein familyTIGR0009) was not present in all considered genomes and was therefore omitted. The amino acid sequences were concatenated and aligned by MAFFT (Katoh et al. 2005). GBLOCKS (version 0.91b) (Castresana 2000) was used to filter out highly variable positions, which resulted in a reduction of the alignment from 47,304 to 42,980 positions in 310 selected blocks, which represents 90% of the alignment positions. A RAxML tree (Stamatakis 2014) with 100 bootstrap replicates was calculated using the CIPRES Science Gateway version 3.3 (Miller et al. 2010).

## Results

### Phenotypic and chemotaxonomic characteristics

**Fig. 2:**
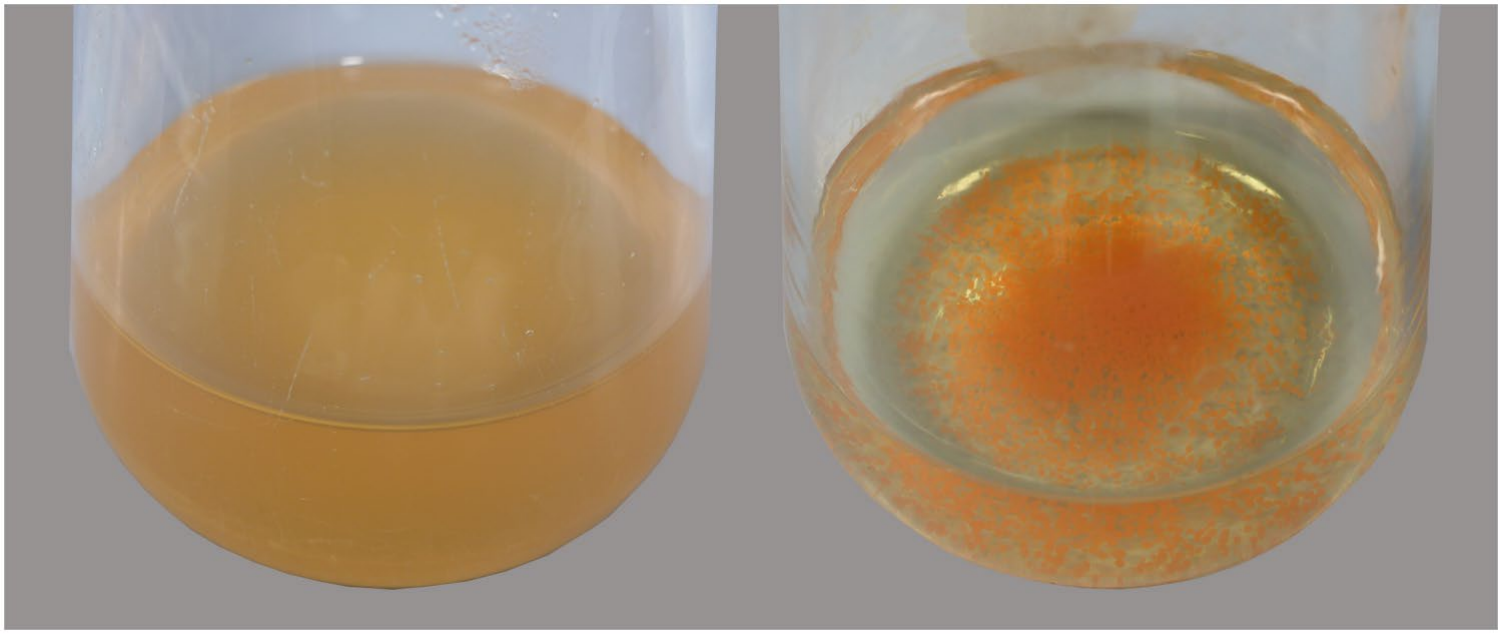
Growth and pigmentation of the two new strains in liquid NSY medium. Left: *Aquirufa lenticrescens* 9H-EGSE^T^; right: *Aquirufa aurantiipilula* 15D-MOB^T^

Table 1 shows phenotypic and chemotaxonomic features of strains 9H-EGSE^T^ and 15D-MOB^T^ and for comparison of the nearest related type strains of the genus *Aquirufa*. Strain 15D-MOB^T^ showed a feature, which was by now unique among all strains of the genus. In liquid medium, the cells formed tiny orange globules (Fig. 2) and were not suspended, as usual for *Aquirufa* strains. This clump-forming made it impossible to get reliable results for the substrate (GEN III) tests and difficult to measure the cell dimensions. The substrate assimilation patterns can be found in Table 1, for not listed substrates, negative results were detected for all stains. Strain 9H-EGSE^T^ assimilated mucic acid beside Tween 40 and glucuronamide, which was not observed for the closely related strains of the genus *Aquirufa*. The patterns of the fatty acids were very similar to the patterns of the closely related type strains (Pitt et al. 2020) but differed by the absence of fatty acid Unknown 14.9591 (identified by mass spectrometry as C_15:1_ω4c) in the cells of strains 9H-EGSE^T^ and 15D-MOB^T^ (Table S1, Online Resource 1 and Table 1). The major respiratory quinone was for both strains MK7, as so far detected for all *Aquirufa* strains, for strain 9H-EGSE^T^ traces of MK8 were identified in addition (Table 1). The results of analyses concerning the polar lipid patterns of strains 9H-EGSE^T^ and 15D-MOB^T^ are given in Online Resource 1, Figure S1. For both strains, phosphatidylethanolamine was detected, as so far for all *Aquirufa* strains and occurred beside unidentified aminophospholipids and lipids, for strain 9H-EGSE^T^ the coloring with molybdenum blue revealed two additional aminophospholipids (Table 1).

**Table 1:** Features of the two new strains and the nearest related type strains of the genus *Aquirufa*

	**1**	2	**3**	4	5	6
Liquid culture (NSY)	Red–orange suspension	Red–orange suspension	Orange beads	Red–orange suspension	Red–orange suspension	Red–orange suspension
Mean cell length (µm)	1.2	1.7	1.9^b^	1.7	1.5	1.6
Mean cell width (µm)	0.5	0.6	0.6^b^	0.5	0.3	0.5
Temperature range for growth (°C)	5–31 (w)	5–32 (w)	5–35 (w)	5–34	5–30 (w)	5–35 (w)
NaCl tolerance (%, w/v)	0–0.1 (w)	0–0.3 (w)	0–0.3	0–0.4	0–0.2 (w)	0–0.4
Assimilation of:						
Pectin	–	+	ND^b^	–	+	+
Tween 40	+	+	ND^b^	w	+	+
Acetoacetic acid	–	w	ND^b^	w	w	w
Glucoronamide	w	w	ND^b^	–	w	w
D-Fructose-6-PO_4_	–	w	ND^b^	–	w	w
Mucic acid	w	–	ND^b^	–	–	–
Dextrin	–	–	ND^b^	w	–	–
Acetic acid	–	–	ND^b^	–	w	–
Sucrose	–	–	ND^b^	–	–	w
D-Mannose	–	–	ND^b^	–	–	w
D-Salicin	–	–	ND^b^	–	–	w
D-Aspartic acid	–	–	ND^b^	–	–	w
Respiratory quinones:						
MK6	–	TR	–	–	TR	–
MK8	TR	–	–	–	–	–
Fatty acid:						
Unknown 14.959^a^	–	2.4	–	4.6	5.0	5.0
Polar lipids:						
Unidentified aminolipids	–	–	–	–	1	1
Unidentified aminophospholipids	3	2	3	3	3	3
Unidentified phospholipids	–	–	–	–	1	–
Unidentified polar lipids	2	4	2	2	2	5

**1**, *Aquirufa lenticrescens* 9H-EGSE^T^; 2, *A. antheringensis* 30S-ANTBAC^T^; **3**, *A. aurantiipilula* 15D-MOB^T^; 4, *A*. *ecclesiirivi* 50A-KIRBA^T^; 5, *A. beregesia* 50C-KIRBA^T^; 6, *A. nivalisilvae* 59G-WUEMPEL^T^. All strains had in common: cell morphology: rods, pigmentation colonies: red, motility on soft agar: + , anaerobic growth: −, major respiratory quinone: MK 7, identified polar lipid: phosphatidylethanolamine. Only the differentiating fatty acid is listed. The whole fatty acid composition of the new strains can be found in Online Resource 1, Table S1. -, negative; + , positive; w, weak; ND, not determined; TR, traces

^a^(identified by mass spectrometry as C_15:1_ω4c)

^b^Difficult/not possible to measure due to clump-forming

Data in columns 2 and 6 (Pitt et al. 2019) and columns 4 and 5 (Pitt et al. 2020) were published previously, but elevated under the same conditions

### Genomic characteristics

Some features of the genomes of strains 9H-EGSE^T^ and 15D-MOB^T^ as well as of the type strains of all *Aquirufa* species and comparisons between them are listed in Table 2, genome sizes and G + C values can be found in Fig. 1. The sizes and G + C values of the genome sequences of the new strains consolidated the impression, that species of *Aquirufa*, which are closely related, have nearly the same genome sizes and G + C values (Fig. 1).

**Table 2:** Genomic traits of the two new strains and all type strains of the genus *Aquirufa*

	**1**	2	**3**	4	5	6	7
IMG ID number	2857132225	2816332120	2857134496	2828879446	2816332124	2816332125	2844599478
gANI with 9H-EGSE^T^ (%)	100	88.6	75.6	75.4	75.4	75.5	75.2
Corresponding average AF (%)	100	87.9	37.5	36.7	35.4	36.6	34.5
gANI with 15D-MOB^T^ (%)	75.6	75.5	100	85.9	86.3	85.7	79.9
Corresponding average AF (%)	37.5	36.5	100	87.3	87.7	88.2	83.5
dDDH with 9H-EGSE^T^ (d_4_, %)	100	36.1	18.7	18.3	18.5	18.4	18.2
dDDH with 15D-MOB^T^ (d_4_, %)	18.7	18.7	100	30.0	31.0	29.6	21.7
Number of homolog genes with 9H-EGSE^T^	**2218**	2011	1829	1816	1798	1813	1790
Number of homolog genes with 15D-MOB^T^	1861	1853	**2674**	2406	2434	2421	2357
Genes predicted for:							
Bacteriorhodopsin (COG5524)	+	+	–	–	–	–	–
β-Carotene 15,15'-monooxygenase (TIGR03753)	+	+	–	–	–	–	–
Synthesis of β-carotene (KEEG map00906)	+	+	+	+	+	+	+
Nitrous oxide reductase (EC:1.7.2.4)	–	–	+	+	+	+	+
Nitrate reductase, assimilatory (EC:1.7.7.2)	–	+	–	+	+	+	–
Nitrite reductase, assimilatory (EC:1.7.1.15)	–	+	–	+	+	+	–
MFS transporter: nitrate/nitrite (COG2223)	–	+	–	+	+	+	–
Catalase-peroxidase EC:1.11.1.21	+	+	+	+	–	–	+
Cytochrome c peroxidase EC:1.11.1.5	+	+	–	–	–	–	–

**1**, *Aquirufa lenticrescens* 9H-EGSE^T^; 2, *A. antheringensis* 30S-ANTBAC^T^; **3**, *A. aurantiipilula* 15D-MOB^T^; 4, *A*. *ecclesiirivi* 50A-KIRBA^T^; 5, *A. beregesia* 50C-KIRBA^T^; 6, *A. nivalisilvae* 59G-WUEMPEL^T^; 7, *A. rosea* Car-16^ T^

**Fig. 3:**
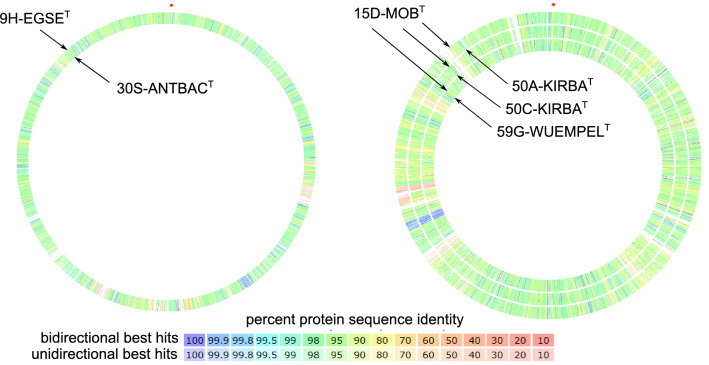
Sequence-based comparison of the genomes of the two new strains with the genomes of the nearest related type strains of the genus *Aquirufa*. Left: *Aquirufa lenticrescens* 9H-EGSE^T^ (outer circle) and *A. antheringensis* 30S-ANTBAC^T^ (inner circle); right: *A. aurantiipilula* 15D-MOB^T^ (in each case outer circle), *A. ecclesiirivi* 50A-KIRBA^T^ (first inner circle), *A*. *beregesia* 50C-KIRBA^T^ (second inner circle), *A. nivalisilvae* 59G-WUEMPEL^T^ (third inner circle). The colors indicate the percentage of the amino acid sequence identity for bidirectional and unidirectional best hits (see scale)

Analyses of the homologous protein-encoding genes (Table 2) showed that strain 9H-EGSE^T^ shared from its 2218 genes 90.7% with the type strain of the nearest relative *Aquirufa antheringensis*, strain 15D-MOB^T^ from its 2674 genes as well around 90% with the type strains of the nearest related species *A. beregesia*, *A. ecclesiirivi,* and *A. nivalisilvae*. The amino acid sequence-based comparison of the new strains with their nearest relatives (Fig. 3) showed in case of strain 15D-MOB^T^ similar patterns in each case of comparison with the three closest related type strains. Nevertheless, the comparisons illustrate genomic differentiation between the strains and their relatives, indicated by low sequence similarity regions and unique genes identifiable by white areas (no similarity) of the circles (Fig. 3). Regarding the specifically contained genes, strain 9H-EGSE^T^ showed genes predicted for synthesis of the complete light-harvesting rhodopsin / retinal system. Interestingly the strain had this feature in common with the type strain of the closest related species *A. antheringensis*, with a sequence similarity for the rhodopsin gene of 95.2% for nucleotides and 99.2% for amino acids. In contrast strain 15D-MOB^T^ and the three related type strains of *A. beregesia*, *A. ecclesiirivi* and *A. nivalisilvae* lacked these genes (Table 2). Both new strains showed like all by now described strains of the genus *Aquirufa* several genes predicted for gliding motility associated proteins and lipoproteins and for cytochrome c oxidase as well as cytochrome cbb-3 oxidase. The new strains had genes presumably encoded for peroxidases comparable with the nearest related strains (Table 2). Table 2 shows as well the calculated gANI with the corresponding average AF values and dDDH percentages. Strain 9H-EGSE^T^ revealed the highest gANI value with the type strain of *A. antheringensis* with 88.6% (AF 87.9%) and the highest dDDH value amounted to 36.1% for the same pairing. Respective strain 15D-MOB^T^ comparisons with the type strain of *A. beregesia* yielded the highest values, gANI 86.3% (AF 87.7) and a dDDH 31%.

### Phylogeny

**Fig. 4:**
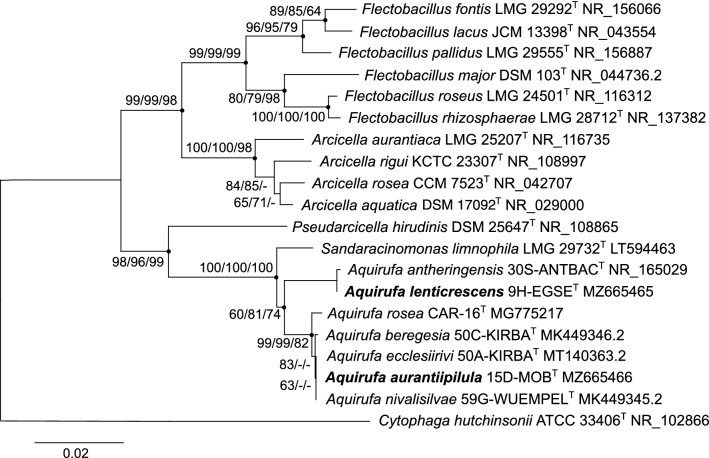
Reconstruction of the phylogenetic position of the investigated strains based on almost full-length 16S rRNA gene sequences (1322 alignment positions). Shown is the neighbor-joining tree. Bootstrap values are shown from left to right for neighbor-joining, maximum likelihood, and maximum parsimony trees calculated with the same sequence set. Bar, 0.02 substitutions per nucleotide position; dots, nodes present in all calculated trees

Both phylogenetic trees (Figs. 1 and 4) showed nearly the same structure, only the position of *Pseudarcicella hirudinis* differed. In the genome-based phylogenetic tree, the cluster comprising the genera *Flectobacillus*, *Arcicella*, and *Pseudarcicella* with genome sizes around 6 Mbp was well separated from the cluster including the genera *Sandaracinomonas* and *Aquirufa* with significant smaller genome sizes of around 3 Mbp (Fig. 1). While the 16S rRNA sequence-based tree could not fully resolve the relationships between the species of the genus *Aquirufa*, the genome-based tree showed the phylogenetic distances of the two new strains to strains of the so far described species (Fig. 1).

## Discussion

As mentioned above, the 16S rRNA sequence similarities between the species belonging to the genus *Aquirufa* are very high, so the new strain 9H-EGSE^T^ showed sequence similarity with *A. antheringensis* of 99.9% and strain 15D-MOB^T^ of 100% with *A. nivalisilvae* and *A. ecclesiirivi* and 99.9% with *A. beregesia*. This circumstance of very high interspecific 16S rRNA gene similarity is well described for several genera (Stackebrandt and Ebers 2006; Hahn et al. 2016). Presumable are here the diversification rates, which lead to species separation higher than the substitution rates of the ribosomal genes. As evidence for a new species serves in this case the gANI or the dDDH value (Chun et al. 2018). For both new strains were the highest determined gANI values with 88.6% and 86.3%, respectively clearly under the accepted threshold of 95–96% utilized to delineate two prokaryotic species (Konstantinidis et al. 2006; Chun et al. 2018). The calculated dDDH values of maximal 36.1% and 31.0%, respectively, underlined this fact, as the accepted threshold is here 70% (Chun et al. 2018). So, strain 9H-EGSE^T^ and 15D-MOB^T^ represent both new species. While most of the phenotypic and chemotaxonomic features of the two new strains resemble the patterns of the so far described *Aquirufa* species, some characteristics were suitable for discrimination (Table 1). Concerning the phenotypic features strain 9H-EGSE^T^ assimilated mucic acid beside Tween 40 and glucuronamide, which was not observed for the closely related strains of the genus *Aquirufa* (Table 1). Strain 15D-MOB^T^ could be distinguished by the phenotypic feature, that cells formed tiny orange globules in liquid medium from all until now described strains of the genus *Aquirufa* and strain 9H-EGSE^T^ (Fig. 2 and Table 1). Concerning the chemotaxonomic traits, the cells of both new strains did not contain the fatty acid Unknown 14.959, as it was found for the closely related type strains as well the patterns of the polar lipids varied slightly (Table 1). Strain 9H-EGSE^T^ differed from all so far described type strains of the genus *Aquirufa* and strain 15D-MOB^T^ by the occurrence of MK8 as respiratory quinone (Table 1) and (Sheu et al. 2020).

The genome-based phylogenetic tree (Fig. 1) in combination with the genomic data in Table 2 revealed some interesting points about the genus *Aquirufa*. The RAxML tree showed two distinct branches of the genus, in which the associated species had similar genome sizes and G + C values in common. This fact and comparisons with the branch length, thus the phylogenetic distances, between the genera *Aquirufa* and *Sandaracinomonas* on the one hand and *Flectobacillus, Arcicella,* and *Pseudarcicella*, on the other hand, suggested that the two main branches of the current genus *Aquirufa* could be regarded as two separated genera. The relatively low AF values of around 35% for genome pairs composed of strains from both *Aquirufa* branches supported this impression. On the other hand, the phenotypic and chemotaxonomic properties of the strains related to the two branches were very similar. Since, besides the 16S rRNA gene similarity, which is here not suitable, clear thresholds for separating genera are lacking this point must be left open. Maybe further isolations of new strains will enhance the impression of two distinct genera or will reveal ‘missing links’ between the two *Aquirufa* branches or between the genera *Aquirufa* and *Sandaracinomonas*. Therefore, we propose here only the establishment of two new species of the genus *Aquirufa*, with the name *Aquirufa lenticrescens* for strain 9H-EGSE^T^ and *Aquirufa aurantiipilula* for strain 15D-MOB^T^.

The occurrence of genes predicted for oxidases and peroxidases suggested that strains 9H-EGSE^T^ and 15D-MOB^T^ occur under aerobic conditions, especially the cytochrome cbb-3 oxidase indicated tolerance to microaerobic conditions (Pitcher et al. 2002). Genes putatively encoding for synthesis of the complete light-harvesting rhodopsin / retinal system in strain 9H-EGSE^T^ suggested a distribution in upper water layers, where enough light is available. The gliding motility, observed in experiments and confirmed by the occurrence of corresponding genes, implicated the association with substrate.

## Description of *Aquirufa lenticrescens *sp. nov. (len.ti.cres’cens. L. masc. adj. *lentus*, slow; L. pres. part. crescens, growing; N.L. part. adj. *lenticrescens*, slow-growing)

Cells form rods, about 1.2 µm long and 0.5 µm wide. Colonies grown on NSY or R2A agar are bright red, in older stages dark red, pigmented, circular, and convex with a smooth surface. Liquid cultures grown in NSY or R2A medium have a red–orange coloring. Cells can move on soft agar. Growth occurs at 5–31 °C and in 0–0.1% (w) NaCl. Cells assimilate Tween 40, weakly assimilate mucic acid and glucuronamide, they do not assimilate the rest of the GEN III MicroPlatesT (Biolog). Major fatty acids are iso-C_15:0_, anteiso-C_15:0_, and summed feature 3 (C_16:1_ω7c / C_16:1_ω6c, identified by mass spectrometry as C_16:1_ω7c). Polar lipids are phosphatidylethanolamine, three unidentified aminophospholipid, and two unidentified polar lipids. Major respiratory quinone is MK7, traces of MK8 occur. The genome of the type strain is characterized by a size of 2.5 Mbp and a G + C content of 42.2 mol%.

The type strain is 9H-EGSE^T^ (= JCM 34077^ T^ = CIP 111926^ T^), which was isolated from Grossegelsee, a small pond, located near the town of Mattsee, Austria.

The accession number of the genome sequence is CP049834 and MZ665465 of the 16S rRNA sequence.

## Description of *Aquirufa aurantiipilula *sp. nov. (au.ran.ti.i.pi’lu.la. L. masc. adj. *aurantius*, orange; L. fem. n. *pilula*, small ball; N.L. fem. n. *aurantiipilula*, orange colored small ball)

Cells form rods, about 1.9 µm long and 0.6 µm wide. Colonies grown on NSY or R2A agar are bright red, in older stages dark red, pigmented, circular, and convex with a smooth surface. Grown in liquid NSY or R2A medium cells accumulate to tiny orange globules. Cells can move on soft agar. Growth occurs at 5–35 °C and in 0–0.3% (w) NaCl. Major fatty acids are iso-C_15:0_, anteiso-C_15:0_, and summed feature 3 (C_16:1_ω7c / C_16:1_ω6c, identified by mass spectrometry as C_16:1_ω7c). Polar lipids are phosphatidylethanolamine, three unidentified aminophospholipid, and two unidentified polar lipids. Major respiratory quinone is MK7. The genome of the type strain is characterized by a size of 3.1 Mbp and a G + C content of 38.0 mol%.

The type strain is 15D-MOB^T^ (= JCM 34078^ T^ = CIP 111925^ T^), which was isolated from Moosbach, a small creek, running through the town Mondsee, near Salzburg, Austria.

The accession number of the genome sequence is JAANOG000000000 and MZ665466 of the 16S rRNA sequence.

## Supplementary Information

Below is the link to the electronic supplementary material.

Supplementary file1 (PDF 134 KB)

## Data Availability

The sequences were available at DDBJ/ENA/GenBank. The accession numbers of the whole-genome sequence of strain 9H-EGSE^T^ are CP049834 and of strain 15D-MOB^T^ JAANOG000000000 of the 16S rRNA gene sequence of strain 9H-EGSE^T^ is MZ665465 and of strain 15D-MOB^T^ MZ665466. All genomes in Table 2 are available in the IMG/MER system (ID numbers see Table 2).

## Abbreviations

NSY medium: Nutrient broth, soytone, yeast extract medium
IMG/MER: Integrated Microbial Genomes and Microbiomes Expert Review
gANI: Whole-genome average nucleotide identity
AF: Alignment fraction
dDDH: Digital DNA–DNA hybridization
*A*: *Aquirufa*
