# Supplementary material for: Aquirufa lenticrescens sp. nov. and Aquirufa aurantiipilula sp. nov.: two new species of a lineage of widespread freshwater bacteria
Source: Arch Microbiol. 2022 Jun 2;204(6):356. doi: 10.1007/s00203-022-02950-6 (PMC9163014; doi:10.1007/s00203-022-02950-6)

Supplementary Information

Archives of Microbiology

Aquirufa lenticrescens sp. nov. and *Aquirufa aurantiipilula* sp. nov., two new species of a lineage of widespread freshwater bacteria

Alexandra Pitt, Ulrike Koll, Johanna Schmidt, Meina Neumann-Schaal, Jacqueline Wolf, Sophia Krausz and Martin W. Hahn

Corresponding author: Alexandra Pitt, Research Department for Limnology,
University of Innsbruck, Mondseestrasse 9, 5310 Mondsee, Austria
email, Alexandra.Pitt@uibk.ac.at

Table S1. Fatty acid composition of strains 9H-EGSE^T (1) and 15D-MOB^T (2).

Only fatty acids with values ≥ 1 % for at least one of the strains were listed. Major fatty acids (> 10 %) were marked with bold letters.

	1	2
$C_{14:0}$	1.4	0.7
$C_{15:1}\omega6c$	2.4	0.9
$C_{16:1}\omega5c$	7.0	8.2
$C_{17:1}\omega6c$	1.7	2.0
iso-$C_{11:0}$	2.0	1.6
**iso-$C_{15:0}$**	**41.4**	**34.9**
**anteiso-$C_{15:0}$**	**15.5**	**13.2**
Iso-$C_{15:0}$ 3-OH	4.5	7.1
Iso-$C_{16:0}$ 3-OH	1.2	0.6
Iso-$C_{17:0}$ 3-OH	1.1	2.5
**Summed feature 3[a]**	**12.0**	**16.6**
Summed feature 4[a]	2.9	3.6
Summed feature 9[a]	2.1	2.6

[a] Summed feature represents a group of fatty acids which could not be separated by GLC and the MIDI system. Summed feature 3, $C_{16:1}\omega7c$, $C_{16:1}\omega6c$ (identified by mass spectrometry as $C_{16:1}\omega7c$); summed feature 4, anteiso-$C_{17:1}$ B, iso-$C_{17:1}$ I (identified by mass spectrometry as Iso-$C_{17:1}\omega5c$); summed feature 9, Iso-$C_{17:1}\omega9c$, 10-methyl $C_{16:0}$ (identified by mass spectrometry as Iso-$C_{17:1}\omega7c$)

Fig. S1. Polar lipid patterns of strains 9H-EGSE^{T} (F21-52) and 15D-MOB^{T} (F21-51).

Right side: coloring with dodecamolybdophosphoric acid, left side: coloring with molybdenum blue.

PE, phosphatidylethanolamine; APL, aminophospholipid; L, lipid

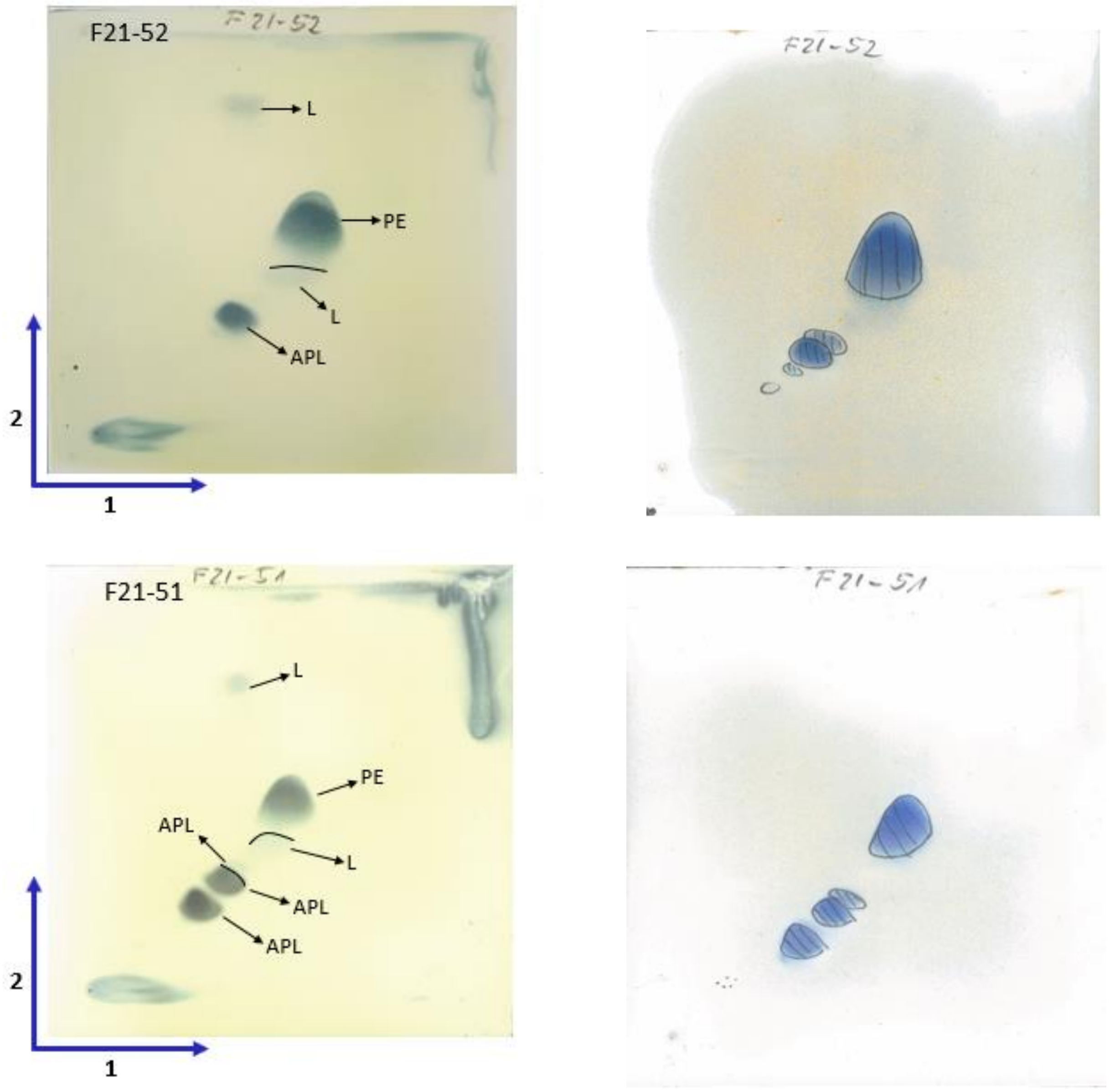

Supplement: Supplementary file 1 — Supplementary file1 (PDF 134 KB) [file 203_2022_2950_MOESM1_ESM.pdf]
